# Complete Resolution of a Recurrent Canine Anal Sac Squamous Cell Carcinoma with Palliative Radiotherapy and Carboplatin Chemotherapy

**DOI:** 10.3390/vetsci4030045

**Published:** 2017-09-14

**Authors:** Antonio Giuliano, Jane Dobson, Sarah Mason

**Affiliations:** Department of Veterinary Medicine, University of Cambridge, Cambridge CB3 0ES, UK; jmd1000@cam.ac.uk (J.D.); sm2164@cam.ac.uk (S.M.)

**Keywords:** anal sac squamous cell carcinoma, carboplatin, radiotherapy

## Abstract

Anal sac squamous cell carcinoma (SCC) is a rare tumor in dogs. Only eight cases have been described in the literature, and previous reports of treatment only describe surgery or palliative treatment with non-steroidal anti-inflammatory drugs. We report a case of a 12-year-old female neutered Labrador with locally advanced anal sac SCC. The dog was treated with four cycles of carboplatin 300 mg/m^2^ and four weekly fractions of 8.5 Gy radiation. The dog achieved a complete response, and one year later the dog is still alive and well with no evidence of tumor recurrence. Radiotherapy in combination with carboplatin chemotherapy was effective in the long-term control of this rare disease.

## 1. Case Report

**Figure 1:**
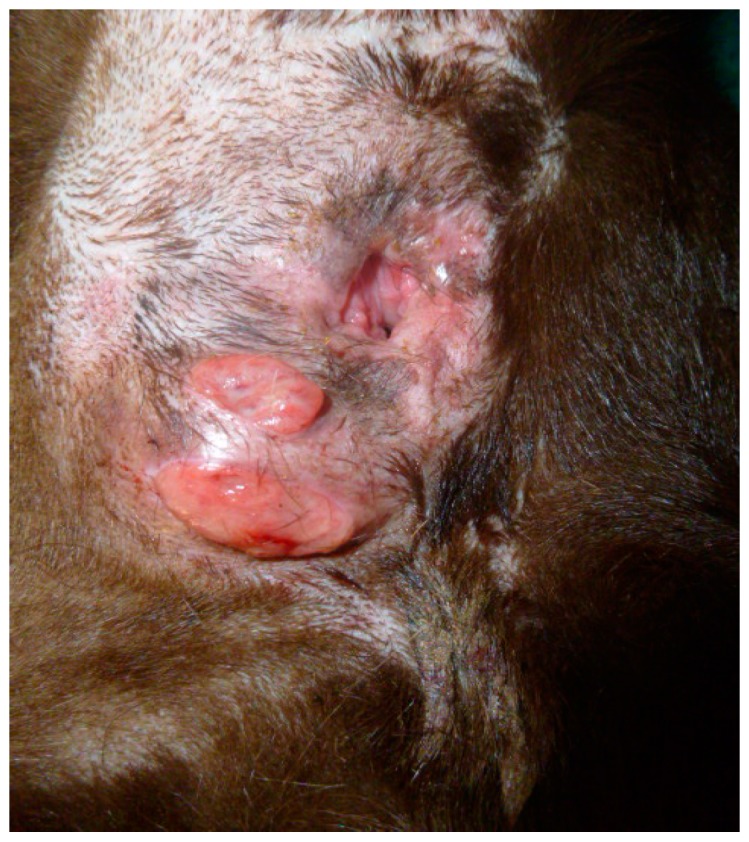
Pre-treatment.

A 12-year-old female neutered Labrador retriever was presented for a mass in the left anal sac. The mass was removed by the referring veterinary surgeon, and a histological diagnosis of anal sac SCC (incompletely resected) was made by a board-certified pathologist. The tumor rapidly recurred less than one month later and the dog was referred for further investigation and treatment. On presentation, the owner reported tenesmus and discomfort in the anal region. A firm, ulcerated mass, approximately 5 cm in diameter, extended over the entire left perianal region, involving slightly more than 50% of the anal circumference (Figure 1). On rectal examination, the mass appeared to be extending/adherent to the rectal wall, but the sacral lymph nodes were palpably within normal limits.

Hematology and biochemistry were within normal limits. Abdominal ultrasound and thoracic radiography revealed no evidence of local or distant metastases. Advanced imaging, computerised tomography (CT) scan was declined by the client for financial reasons. 

Due to its rapid recurrence following surgery, its location, and extent, the tumor was considered inoperable. The prognosis was considered to be very poor.

**Figure 2:**
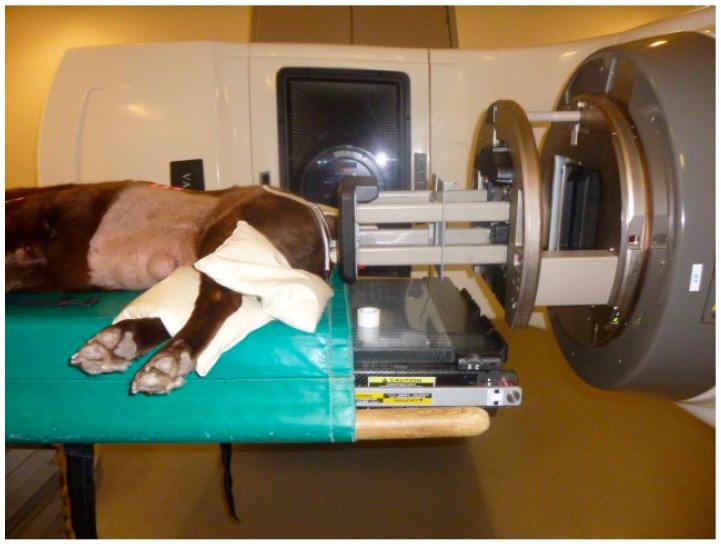
Position of the patient for the radiotherapy treatment.

A palliative course of radiotherapy was prescribed and the dog received four, once weekly 8.5 Gy fractions of 12 MeV electrons (34 Gy in total) delivered by a Varian Clinac 2100 IX DMX (Varian, Palo Alto, CA, USA). The patient was placed in right lateral recumbency with the beam perpendicular to the tumor in an “en face” position (Figure 2). The radiation field and dose were calculated manually to minimize the radiation exposure of rectum and anus. A rectangular 8 × 6 cm electron cut out was selected and a 1 cm bolus to the skin was applied to improve the surface dosing. Chemotherapy with carboplatin was also administered and the patient received 300 mg/m^2^ every three weeks (weeks one and four of radiation, and two subsequent doses, at three-week intervals). Complete blood counts were performed prior to each carboplatin administration and were within normal limits. The patient was also continued on 0.1 mg/kg of oral meloxicam (Metacam; Boehringer-Ingleheim, Bracknell, Berkshire, UK) once a day as previously prescribed by the referring veterinary surgeon. Amoxicillin/clavulanate (Clavaseptin; Vetoquinol, Buckingham, Buckinghamshire, UK) 15 mg × kg was administered twice a day for two weeks for the treatment of secondary infection of the ulcerated tumor mass. The anal mass reduced in size during the first four weeks of radiotherapy/chemotherapy treatment and no acute side effects from the chemotherapy and radiotherapy were observed.

**Figure 3:**
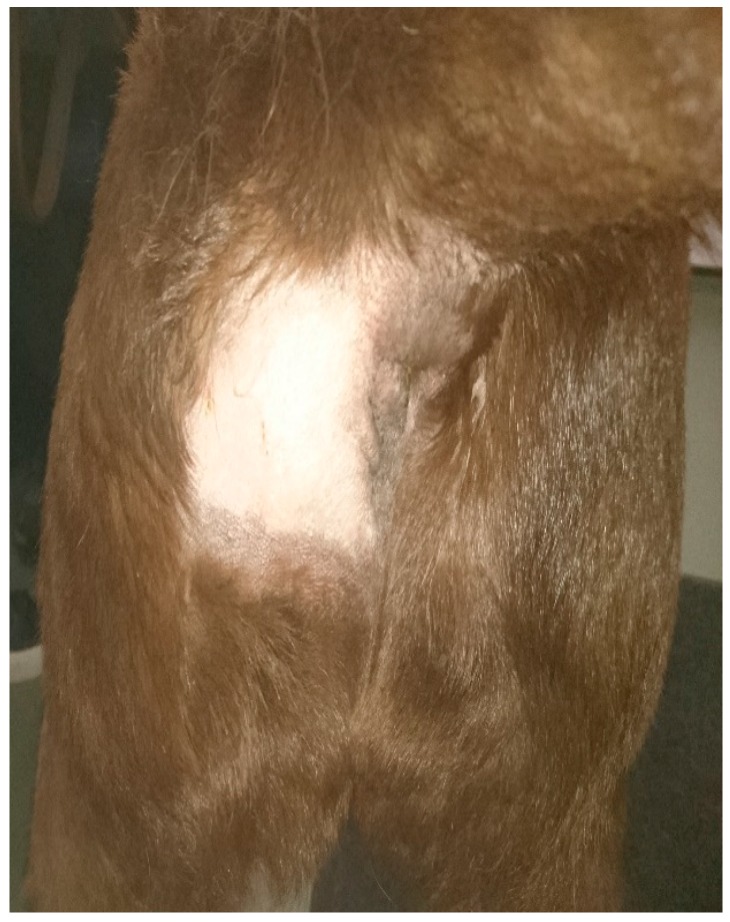
Six weeks post treatment.

Two weeks following the final radiotherapy treatment, the tumor had completely regressed with no further signs of the tumor on rectal palpation (Figure 3). The dog was regularly rechecked every two to three months for signs of recurrence. One year following diagnoses the dog was alive and well, with no evidence of local recurrence or sub-lumbar lymph node metastases on physical examination. Radiation toxicity was limited to alopecia of the treated skin. The client declined re-staging.

## 2. Discussion

SCC is a relatively common tumor arising from the oral mucosa of the mouth and the subungual epithelium [1,2,3]. Less commonly in the dog, SCC develops in the skin, with most common locations reported to be the scrotum, nasal planum, perineum, and limbs [4].

Anal sac squamous cell carcinoma (SCC) is a rare tumor in dogs. Only eight cases have been previously published in the literature, with no reports describing response to chemotherapy and radiotherapy. In the five cases reported by Esplin et al., 2003 [5] it was suggested that anal sac SCC has an aggressive biological behavior and carries a poor prognosis. More recently, Mellet et al., 2015 [4] reported that palliative treatment with meloxicam in three dogs resulted in a survival time of 150, 79, and 210 days, respectively.

In dogs, the combination of radiotherapy with carboplatin chemotherapy has been reported to have some benefit in the treatment of tonsillar and oral SCC [2,6,7,8,9,10]. This evidence supported our therapeutic rationale in this case. Given the extent of the disease at presentation, this bi-modal therapeutic approach was thought to offer the best chance of palliation and patient survival.

For definitive or curative intent radiotherapy of pelvic tumors, a fractionated photon protocol is indicated to achieve a dose at depth and minimize the risk of late toxicity to the rectum [11]. Palliative radiation protocols generally deliver total doses of around ~30 Gy and have been reported for locally extensive and metastatic anal sac adenocarcinoma [11,12,13]. The risk of late side effects of the hypofractionated radiotherapy schedule to the pelvic region, in particular chronic colitis, anal/rectal stricture, and rectal perforation/fistula, were discussed with the owner. However, because such side effects usually manifest many months to years after treatment, and this patient was considered unlikely to achieve long-term survival, these were accepted. The owner’s main objective was quality of life in the short term and relief of the clinical signs. Electrons were chosen over photons due to the rapid dose fall-off at depth (for 12 MeV, less than 10% of the applied dose at 6 cm), thus reducing the risk of acute and late side effects on pelvic organs including the anus and rectum. The former (acute effects) were particularly important given that short-term quality of life was the main aim of the treatment. The addition of a 1-cm bolus resulted in the ulcerated tumor mass and adjacent skin surface receiving 94% of the applied dose. This effectively resulted in the tumor mass receiving 95%+ of the applied dose to a depth of 3 cm. In the acute, short-term this resulted in mild erythema of the skin and latterly total alopecia, although the skin remained supple and healthy.

A CT scan of the abdomen was not performed, so it could be possible that a potential enlargement of the pelvic lymph nodes were missed at the abdominal ultrasound examination. The same could be said for metastatic screening of the lungs by thoracic radiography rather than by CT. However, due to the lack of lymphadenopathy and/or metastases at the following rechecks, this appears unlikely.

Carboplatin is a radiation sensitizer and combined therapy has been reported to improve outcomes in people with head and neck SCC [14], but this combination can also result in increased acute toxicity in people undergoing curative intent high-dose treatments [15] and this may also be the case in dogs. In the authors’ experience, this rarely manifests clinically in dogs, particularly with hypofractioned radiotherapy protocols. 

Late side effects of chemo-radiation combinations are reported in around 40% of human patients treated for head and neck cancer [16]. Late effects of chemo-radiation combinations have not been widely reported in dogs, but 39% of dogs treated with photon beam radiotherapy to the pelvis experience late side effects [17,18]. The incidence of late side effects of dogs treated with electron beam pelvic hypofractioned radiotherapy is not known. In our case, it is not possible predict the dose that was delivered to the pelvic organs because computer planning was not performed. The role of the dose effect of both the radiation and chemotherapy has not been explored and indeed, as the mechanism of increased toxicity is poorly understood, this is challenging.

Our patient did not develop any late side effects apart from complete alopecia remaining one year following the treatment. It is still possible that more serious late side effects could manifest in the future.

It is our hypothesis that the durable response achieved in this case resulted from the combination of radiation and carboplatin through the radiation-sensitizing actions of the latter. However, it is not possible to know which treatment the tumor actually responded to.

Complete and durable response of gross disease to carboplatin in combination with piroxicam has been previously reported for oral non-tonsillar SCC [19]. However, due to the lack of response to the previous treatment with Non steroidal anti-inflammatory drugs (NSAID) in this case, a combination of carboplatin and radiotherapy is the most likely explanation for tumor response.

## 3. Conclusions

We report a case of complete response with long-term tumor control and survival in a dog with locally recurrent, advanced, and inoperable anal sac SCC following treatment with hypofractioned palliative radiotherapy and carboplatin chemotherapy. The treatment was successful and the patient is alive and well, without signs of recurrence of the disease one year later. Combinations of carboplatin and radiotherapy could be useful in the treatment of anal sac SCC, but the risks of late side effects need to be evaluated, particularly in view of the possible long-term control of the disease. A well-designed, highly powered study would be required to investigate the efficacy of carboplatin and radiotherapy for this uncommon tumor.
